# Hysteresis as an Implicit Prior in Tactile Spatial Decision Making

**DOI:** 10.1371/journal.pone.0089802

**Published:** 2014-02-26

**Authors:** Sabrina D. Thiel, Sebastian Bitzer, Till Nierhaus, Christian Kalberlah, Sven Preusser, Jane Neumann, Vadim V. Nikulin, Elke van der Meer, Arno Villringer, Burkhard Pleger

**Affiliations:** 1 Department of Neurology, Max Planck Institute for Human Cognitive and Brain Sciences, Leipzig, Germany; 2 Clinic for Cognitive Neurology, University Hospital Leipzig, Leipzig, Germany; 3 Mind-Brain Institute and Berlin School of Mind and Brain, Charité – University Medicine Berlin and Humboldt-University, Berlin, Germany; 4 Leipzig University Medical Center, IFB Adiposity Diseases, Leipzig, Germany; 5 Department of Psychology, Faculty of Mathematics and Natural Sciences II, Humboldt-University, Berlin, Germany; 6 Neurophysics Group, Department of Neurology, Campus Benjamin Franklin, Charité - University Medicine Berlin, Berlin, Germany; 7 Centre for Cognition and Decision Making, National Research University Higher School of Economics, Moscow, Russia; University of California, Davis, United States of America

## Abstract

Perceptual decisions not only depend on the incoming information from sensory systems but constitute a combination of current sensory evidence and internally accumulated information from past encounters. Although recent evidence emphasizes the fundamental role of prior knowledge for perceptual decision making, only few studies have quantified the relevance of such priors on perceptual decisions and examined their interplay with other decision-relevant factors, such as the stimulus properties. In the present study we asked whether hysteresis, describing the stability of a percept despite a change in stimulus property and known to occur at perceptual thresholds, also acts as a form of an implicit prior in tactile spatial decision making, supporting the stability of a decision across successively presented random stimuli (i.e., decision hysteresis). We applied a variant of the classical 2-point discrimination task and found that hysteresis influenced perceptual decision making: Participants were more likely to decide ‘same’ rather than ‘different’ on successively presented pin distances. In a direct comparison between the influence of applied pin distances (explicit stimulus property) and hysteresis, we found that on average, stimulus property explained significantly more variance of participants’ decisions than hysteresis. However, when focusing on pin distances at threshold, we found a trend for hysteresis to explain more variance. Furthermore, the less variance was explained by the pin distance on a given decision, the more variance was explained by hysteresis, and vice versa. Our findings suggest that hysteresis acts as an implicit prior in tactile spatial decision making that becomes increasingly important when explicit stimulus properties provide decreasing evidence.

## Introduction

Perceptual decision making is the act of evaluating available sensory information in order to choose one option from a set of alternatives [1]. The selected alternative depends on a variety of factors such as the stimulus inherent properties, task instructions, attention, or motivation [2]. In addition to these explicit factors, implicitly accumulated and integrated information from past encounters form an internal prior which is regarded as an essential ingredient influencing the decision process [3]. In the present study we investigated whether hysteresis, which describes the stability of a percept across successive trials [4] and which is known to influence threshold assessments [5], also acts as such an implicit prior in spatial tactile decision making, leading to the stability of decisions across successively presented stimuli with different stimulus properties (i.e., decision hysteresis).

Most evidence on how the brain evaluates somatosensory stimuli for perceptual decision making has originated from studies using a delayed discrimination task. This task is now widely used because of the unique possibility to accurately separate crucial decision-related sub-processes over time [6]–[8]. More recent human studies have applied this task to investigate the impact of internal prior knowledge on decision behaviour [9], [10]. As a type of prior knowledge, the time-order effect [11]–[13] describes the interaction of stimulus frequency and presentation order that causes a systematic bias in response accuracy and reaction time, although all other task properties are held constant. This suggests that preceding stimuli form an average representation of the stimulus set and implicitly bias the first stimulus of a pair during its encoding and maintenance [9], [10].

Although known for decades, to date, only few studies have examined the relevance of such implicitly formed prior knowledge for perceptual decision making through additionally addressing possible interactions with explicit decision-relevant factors such as stimulus properties. Karim and colleagues emphasized the profound influence of the time-order effect on decision making in their recent study involving participants in the delayed discrimination task [9]. In comparison to three explicit factors, namely task difficulty, stimulus noise, and task instructions, the authors showed that the implicit time-order effect exerts a strong influence on participants’ decisions while being largely independent from any of the three explicit factors of interest.

In the present study we focused on another well-known internal prior based on the stimulus history, namely hysteresis. In the context of perception, hysteresis describes the persistence of an initial percept despite a change in stimulus properties which favors an alternative interpretation [4]. When determining a perceptual threshold, hysteresis explains the difference in stimulus value depending on the direction of stimulus contrast: A degradation of stimulus intensity from initially perceivable to non-perceivable leads to a drop out of the percept at a lower stimulus intensity compared to gradually increasing the intensity from low to high [14]. Perceptual hysteresis thus reflects the non-linear interaction of a percept with stimulus intensity. To account for this interaction when determining a perceptual threshold, the staircase procedure was introduced [5].

Until now, a large body of research has investigated perceptual hysteresis in the visual system applying stimuli either dynamically or statically. For example, Gepshtein, as well as Schwiedrzik and colleagues presented static dot lattices that favor either a left or right tilted orientation depending on the interdot distance. They found that the percept of the first bistable dot lattice guided the percept of the following multistable dot lattice [15], [16].

In addition to provoking hysteresis by multistable stimuli, a large number of studies chose a dynamic approach to elicit and explore the influence of hysteresis. In these studies, stimuli were presented in ascending and descending orders (i.e., staircase procedure). Thus, an impact of hysteresis was demonstrated for motion perception [17], [18], object recognition [14], [19], spatial frequency integration [20], number discrimination [21], as well as perception of emotional expressions [22] and categorisation of ambiguous sentences [23]. Additionally, for the motor system, it has been shown that hysteresis affects grasping behavior [24] and motor planning [25]. Together these studies suggest that in situations providing weak or ambiguous information, prior knowledge complements the decision process. However, applying either descending and ascending orders of stimulus intensities or restricting the stimulus range to highly ambiguous ones may also constrict a broader view of hysteresis like in everyday life situations where such a biased stimulus occurrence is rather rare. This opens the question whether hysteresis plays a role in situations when stimuli occur unbiased.

In the present study we aimed to investigate the influence of hysteresis on tactile spatial decision making when stimuli are presented in a fully balanced and hence unbiased way with regard to the trial-to-trial order of pin distances. This approach is different to previous studies (see above) which investigated different perceptions provoked by the same sensory stimulus, but with different stimulus histories, whereas we were interested in same decisions across successively presented different stimuli (i.e., decision hysteresis). Based on such a presentation schema, we define decision hysteresis as the persistence of a decision across successively presented stimuli with changing stimulus properties.

We asked (i) if hysteresis impacts tactile decision making, (ii) how strongly hysteresis influences participants’ decisions, a) at different pin distances and, b) compared to explicit stimulus property (in the context of our study: the applied pin distance), and (iii) which factors determine the magnitude of hysteresis? To this end, we utilized a variant of the classical 2-point discrimination task, which is the standard instrument for assessing tactile spatial accuracy. We assessed decision-to-decision re-occurrence of same perceptual decisions for successive trials by means of conditional probabilities. We compared the probability to detect a distance between two pins on the current trial given that the same or a different decision (i.e., no distance between pins detected) occurred on the previous trial. To investigate the magnitude of the influence of hysteresis on perceptual decisions, we determined the amount of variance explained, which we next compared with the variance explained by pin distances as an explicit decision criterion.

## Materials and Methods

### Participants

Twenty-six healthy participants (mean age 26 years, SD ±2.6 years, 15 females) took part in this study. All participants were right handed and reported no history of neurological or psychiatric disorder. The study was approved by the Ethical Committee of the University of Leipzig and conducted according to the Human Subjects Guidelines of the Declaration of Helsinki. All participants gave written informed consent and were reimbursed for participation.

### 2-point Discrimination Task

We used a variant of the classical 2-point discrimination task. The tactile spatial discrimination threshold was assessed on the right index fingertip using two plastic pins with cone-shaped heads driven by a piezo-electric stimulation device (Piezo-Electric Stimulation Device, QuaeroSys, Schotten, Germany). The computer controlled piezo-electric device was operated by the Presentation software package (version 14.9, 03.08.11, Neurobehavioral Systems, Inc., Albany, California, United States). In a two-alternative forced-choice design, we applied seven pin distances (0.7, 1.0, 1.3, 1.6, 1.9, 2.2, and 2.5 mm) with a stimulation duration of 1 s. Participants had to decide if they felt a distance between the pins or not. With the unstimulated left hand they indicated their decision with a button press. Participants were instructed to concentrate on their stimulated right index finger and to respond quickly, but as accurately as possible. We pseudo-randomized the pin distance order in a fully balanced fashion to exclude biases in the ordering (i.e., every pin distance appeared in succession with every other pin distance including itself). Participants performed five sessions. Each session consisted of 98 trials (14 repetitions per pin distance). To avoid movements during the task, the participant’s right index finger was fixed with tape on the stimulation device. Participants were comfortably seated in front of a screen signalling the start and end point of the experiment. During the experiment participants wore earplugs and headphones to prevent them from hearing sounds produced by the stimulator that may have affected their attention. After the experiment, participants completed a questionnaire asking them if they had used a specific strategy when deciding on the presented pin distances.

### Tactile Spatial Discrimination Threshold

**Figure 1:**
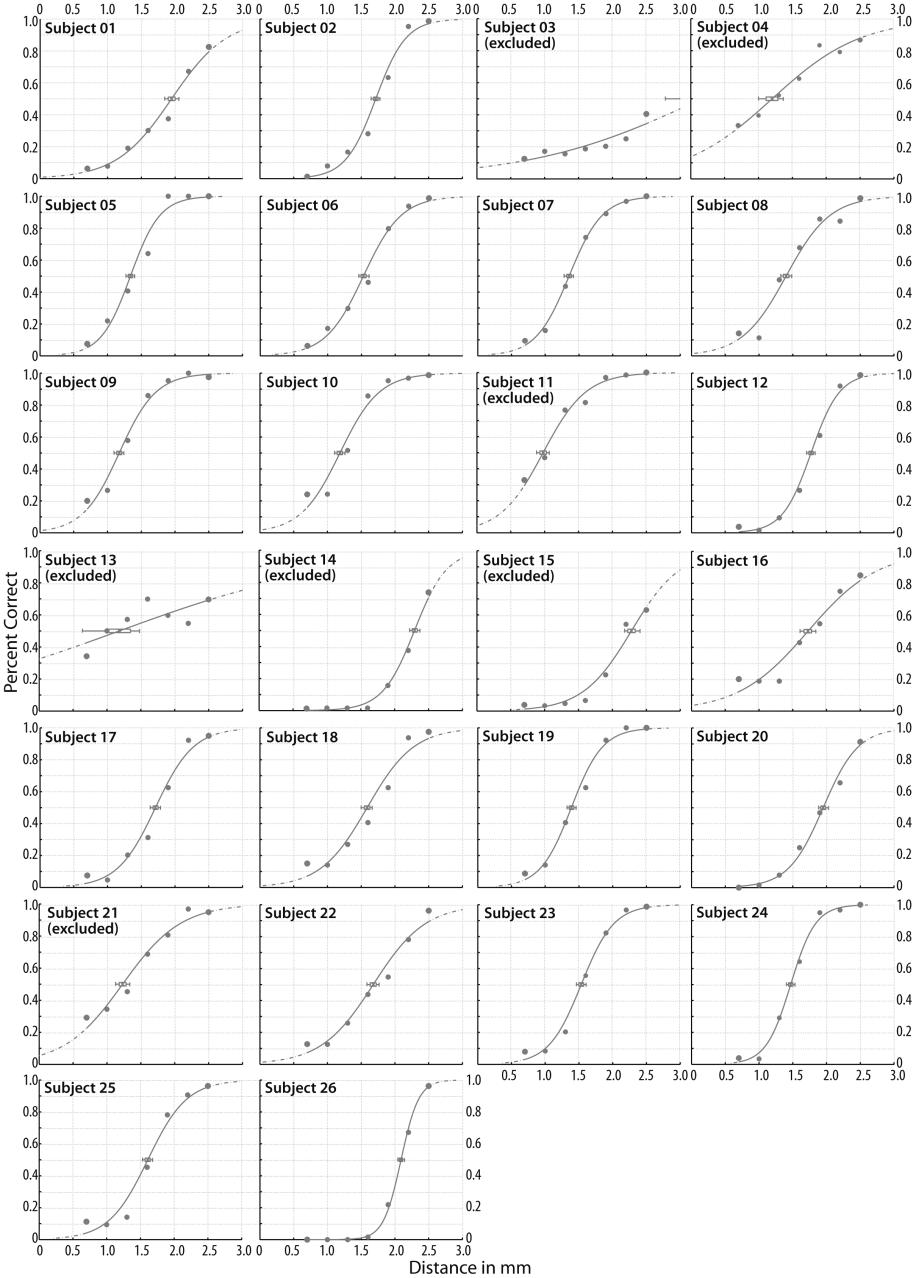
Individual psychometric functions of the 2-point discrimination task. The figure shows individual psychometric functions for each of the 26 participants. The percentages of ‘distance felt’ answers across pin distances (depicted as dots) were fitted with a binary logistic regression and the spatial discrimination threshold was defined as the pin distance closest to the 50% crossing of the fitted sigmoid curve. Error bars are confidence limits derived from bootstrapping 1999 curve fitting simulations. Subjects 3, 4, 11, 13, 14, 15, and 21 were excluded from further analyses (see Materials and Methods).

We calculated psychometric functions using the psignifit toolbox version 2.5.6 for Matlab (see http://bootstrap-software.org/psignifit/) offering the maximum-likelihood method by Wichmann and Hill [26]. The percentages of ‘distance felt’ answers across increasing pin distances were fitted using a binary logistic regression (see e.g., [27]–[29]). The spatial discrimination threshold was defined as the pin distance closest to the 50% crossing of the fitted sigmoid curve. First, we estimated the discrimination threshold for each participant and each of the five sessions to assess stability of discrimination thresholds. Then, we estimated the discrimination threshold of the fitted binary logistic regression for pooled sessions which we used for further analyses (see Figure 1 for individual psychometric functions). Subsequent analyses were carried out for pin distances grouped according to the individual discrimination threshold (pin distance group ‘threshold’), the next two larger (‘threshold+1’, ‘threshold+2’) and the next two smaller pin distances (‘threshold−1’, ‘threshold−2’). We only included participants whose discrimination performance covered the full range of the applied pin distances (i.e., not more than 30% of ‘distance felt’ answers for the smallest distance 0.7 mm and more than 70% ‘distance felt’ answers for the largest pin distance 2.5 mm, respectively). Accordingly, data from seven participants were excluded and group analyses were based on the remaining 19 participants (see Figure 1 for psychometric functions of subjects which were excluded and included). As the maximum trial length was set to 2400 ms, trials with reaction times slower than 2400 ms were discarded from group analyses (out of 2170 trials from 19 participants, 109 trials in 9 participants were discarded).

### Conditional Probabilities

To test if prior information (i.e., the preceding decision) influenced the decision on the current trial, we computed conditional probabilities as follows:
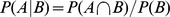
where ‘A’ represents the decision on the current trial and ‘B’ the decision on the preceding one. This allowed us to assess how often ‘distance felt’ decisions on the current trial (‘A’) occurred following either a ‘no distance felt’ (different subsequent decisions) or a ‘distance felt’ (same subsequent decisions) decision on the preceding trial (‘B’).

To assess the main effect of decision re-occurrence, we compared same versus different decisions across the five different pin distances presented at the current trial (‘A’). To account for differences in trial numbers, we applied a weighted averaging approach to each pin distance for each participant. First, we computed conditional probabilities for each possible combination of current and preceding pin distances separately (i.e., for the current distance ‘threshold−2’ following the preceding distance ‘threshold−2’, for the current distance ‘threshold−2’ following the preceding distance ‘threshold−1’, for the current distance ‘threshold−2’ following the preceding distance ‘threshold’, etc.). Next, we determined a weighting factor by dividing the number of trials for each combination of preceding and current pin distances by the number of trials for the current distance. Conditional probabilities were then multiplied by this weighting factor and pooled across the different preceding distances. Finally, we applied the weighted averaging approach to pool across the five current distances. The weighted probabilities for the two possible decision sequences (i.e., same decision versus different decision) were statistically compared using the Wilcoxon signed rank test.

For post-hoc analyses, we compared same versus different subsequent decisions for each of the five current pin distances separately. As for the main effect, probabilities of the two decision sequences (i.e., same decisions versus different decisions) were weighted according to each participant’s number of trials and pooled across different preceding distances. Weighted conditional probabilities for each of the five current distances were statistically compared using the Wilcoxon signed rank test. We corrected *p*-values for the number of tests using the Bonferroni-Holm procedure.

We also looked at the decision history to further assess the number of decisions that correlated with the decision on the current distance. To this end, we calculated conditional probabilities for the decision ‘distance felt’ on the current distance in combination with either the same (i.e., ‘distance felt’) or a different decision (i.e., ‘no distance felt’) for each of the previously presented pin distances for up to 20 distances back in stimulus history. The difference between the conditional probabilities of same and different decisions (i.e., *delta* = same - different) can be interpreted as a correlation between two decisions, with no correlation for *delta* = 0. Assuming a smooth decline in correlation between decisions over the past, we investigated the mean difference in conditional probabilities of same and different decisions (i.e., *delta*) using a nonparametric regression. In particular, we used a Gaussian process model to predict *delta* from the time lag between decisions [30]. We chose the standard squared exponential covariance function in combination with a Gaussian likelihood and fitted hyperparameters to the data across participants by maximizing the Gaussian process marginal likelihood using the Matlab GPML toolbox (http://mloss.org/software/view/263/). The fitted hyperparameter values were *sn* = 0.08, *ell* = 6.08, and *sf2* = 0.04. They can be interpreted as follows: *sn* is the estimated standard deviation of *delta* across participants, *ell* is the characteristic length scale of the Gaussian process and describes the smoothness of the fitted regression curve in terms of time lags. *sf2* is the variance of *delta* when the variability of participants has been accounted for.

To test if the preceding pin distance alone influenced the decision on the current pin distance we computed conditional probabilities where ‘A’ represents the decision on the current pin distance and ‘B’ the preceding pin distance. This enabled us to assess how often ‘distance felt’ decisions to currently presented pin distances (‘A’) occurred in combination with each of the preceding pin distances (‘B’). Then we computed five one-way ANOVAs, one for each of the five current pin distances.

### Explained Variances and Permutation Test

We quantified the influence of the current pin distances (i.e., explicit decision factor) as well as the influence of hysteresis (i.e., implicit decision factor) on decisions by means of explained variances. To this end, for each participant we analysed how well a decision could be predicted either from the current pin distance or the previous decision, respectively. We report the fraction of variance explained as computed from corresponding sums of squared errors of the form
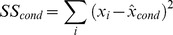
where 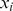 is the decision on trial *i* and 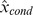 is the mean of decisions, that is the predictor. With this model we (i) estimated the total sum of squares (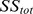), where 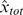 is the mean of decisions across all trials, (ii) the residual sum of squares for decisions based on the current pin distance (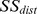), where 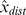 is the mean of decisions across trials for the pin distance presented at trial *i* and (iii) the residual sum of squares for decisions based on the current pin distance and previous decision (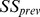), where 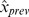 is the mean of decisions across trials for the pin distance presented at trial *i* and the decision at trial *i*−*1*. Based on these computations, the fraction of variance explained by hysteresis is
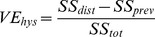
and the fraction of variance explained only by presented pin distances is
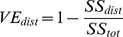



This allowed us to compute both factors’ explained variances (i.e., hysteresis and pin distance) across all five current and preceding pin distances, and for each of the five current and preceding pin distances separately (i.e., for ‘threshold−2’, ‘threshold−1’, ‘threshold’, ‘threshold+1’, ‘threshold+2’). To test statistical significance of explained variances either of pin distances or hysteresis against random distribution, we permuted individual data 10,000 times and calculated explained variances for each permutation. To test the significance of explained variances across participants on the group level, permutations were pooled across participants and compared to the observed variances with the Wilcoxon rank sum test.

First, we compared the amount of variance explained by hysteresis and the variance explained by pin distances between the current pin distance ‘threshold’ and the other four current pin distances (i.e., for ‘threshold’ versus ‘threshold−2’, ‘threshold’ versus ‘threshold−1’, etc.) by means of the Wilcoxon signed rank test. To account for the number of tests we corrected *p*-values with the Bonferroni-Holm procedure.

To directly compare the explained variance of pin distances and hysteresis, we again used the Wilcoxon signed rank test. First, we compared explained variances across all five current and preceding pin distances. Then, in post-hoc tests, we compared explained variances for each of the five current and preceding pin distances. For latter comparisons, we report Bonferroni-Holm corrected *p*-values to account for the number of tests.

### Correlation Analysis

To test whether the variance explained by decision hysteresis related directly to the variance explained by pin distance we used the Spearman rank correlation analysis.

Data was analysed with Matlab (version 2007b, Mathworks) and PASW 18.0 Statistical Package (SPSS, Inc., Chicago, Illinois).

## Results

To examine stability of discrimination thresholds we used a repeated measures ANOVA that revealed no significant changes across all five sessions: *F*(1, 4) = 1.727, *p = *0.153.

### Conditional Probabilities

**Figure 2:**
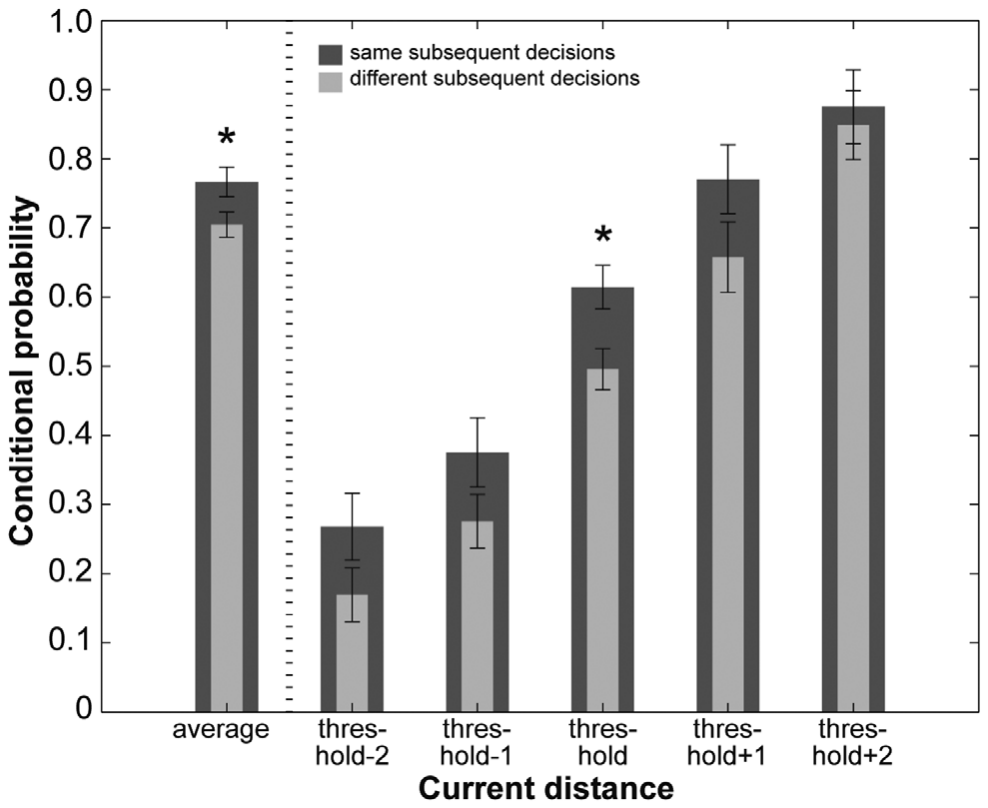
Conditional probabilities for same and different subsequent perceptual decisions. Bar graphs show conditional probabilities for the decision ‘distance felt’ in combination with either the same (dark gray bars) or a different (light gray bars) decision on the preceding trial. The left panel shows probabilities across all five current and preceding pin distances; the right panel shows probabilities for each of the currently presented pin distances in combination with all other pin distances. Whiskers represent the standard error of the mean. Significant differences between same and different decisions are denoted with a star for *p*<0.05. See also Table 1.

We compared conditional probabilities for same and different subsequent decisions across all current pin distances. The Wilcoxon signed rank test revealed a significant difference with higher probabilities for same than for different decisions: *Z* = −2.05, *p*<0.05, see left panel of Figure 2 and Table 1. Post-hoc pairwise comparisons for each of the five current pin distances yielded a significant difference for ‘threshold’ trials indicating a higher probability for same than for different decisions: Wilcoxon signed rank test, *Z* = −2.62, *p*<0.05. For the other four current pin distances ‘threshold−2’, ‘threshold−1’, ‘threshold+1’, and ‘threshold+2’ we only found a pattern towards the same direction (i.e., higher probability for same decisions), see right panel of Figure 2 and Table 1.

**Table 1:** Wilcoxon signed rank tests for conditional probabilities of same versus different subsequent decisions.

Current Pin Distance	Decision ‘same’Mean ± SEM	Decision ‘different’Mean± SEM	WSR *z*-value	WSR *p*-value
Pooled current pin distances	0.77±0.02	0.70±0.02	*Z* = −2.05	*p*<0.05
‘threshold−2’	0.27±0.05	0.17±0.04	*Z* = −1.73	*p* = 0.091
‘threshold−1’	0.38±0.05	0.28±0.04	*Z* = −1.77	*p* = 0.153
‘threshold’	0.61±0.03	0.50±0.03	*Z* = −2.62	*p*<0.05
‘threshold+1’	0.77±0.05	0.66±0.05	*Z* = −1.89	*p* = 0.233
‘threshold+2’	0.88±0.05	0.85±0.05	*Z* = −1.78	*p* = 0.227

WSR: Wilcoxon signed rank test.

**Figure 3:**
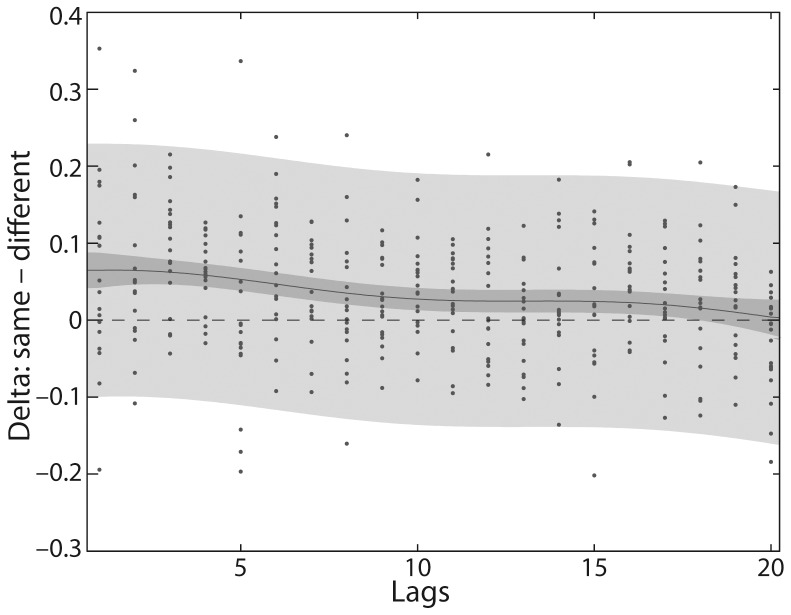
Nonparametric regression analysis for same and different decisions for 20 time lags. Each point shows *delta,* that is, the conditional probability for the decision ‘same’ minus the conditional probability for the decision ‘different’ for each participant. Probabilities for ‘same’ and ‘different’ decisions were computed for the decision on the current distance in combination with a previous decision up to a lag of 20. The solid line shows the regression line and represents the mean values of *delta* for the different lags as inferred from the data of the participants. The light gray shading indicates the region of two standard deviations around the regression line and includes the estimated variability across subjects. The dark gray shading indicates the variability within two standard deviations of the posterior *delta* values that is left when the variability across subjects is accounted for. See Materials and Methods for details.

Moreover, we examined for how many decisions back in the decision history a correlation between current and previous decisions is traceable. A corresponding nonparametric regression analysis revealed a small positive correlation between the current and preceding decision as far back as to the 17^th^ preceding decision once intersubject variability was accounted for (see Figure 3).

We further tested whether the preceding pin distance influenced the decision on the current pin distance. For each of the five current pin distances we computed a one-way ANOVA which yielded no significant effect: ‘threshold−2’: *F*(1, 4) = 0.728, *p = *0.576; ‘threshold−1’: *F*(1, 4) = 1.293, *p = *0.287; ‘threshold’: *F*(1, 4) = 0.707, *p = *0.526; ‘threshold+1’: *F* (1, 4) = 1.005, *p = *0.411; ‘threshold+2’: *F*(1, 4) = 2.567, *p = *0.106).

### Explained Variances

To quantify the contribution of either pin distance or hysteresis to participants’ decisions we determined the amount of variance explained by each of these two factors. We compared explained variance of pin distances and of decision hysteresis to the respective permutation distributions (see Materials and Methods ‘*explained variances and permutation test*’) and found that observed explained variances significantly differed (see Table 2 for an overview).

**Table 2:** Descriptive statistics and statistics for Wilcoxon signed rank tests for explained variance of pin distances and decision hysteresis.

	Explained variance of pin distances				Explained variance of decision hysteresis					
Current PinDistance	Mean ±SEM (%)	Range (%)	WRS*z*-value	WRS*p*-value	Mean ±SEM (%)	Range (%)	WRS*z*-value	WRS*p*-value	WSR*z*-value	WSR*p*-value
Pooled currentpin distances	34,0±3.7	7.1–64.9	*Z* = −7.51	*p*<0.0001	4.9±0.9	0.9–15.0	*Z* = −4.21	*p*<0.0001	*Z* = −3.78	*p*<0.001
‘threshold−2’	52.1±5.3	22.6–100.0	*Z* = −6.90	*p*<0.0001	3.2±0.9	0.0–15.1	*Z* = −2.81	*p*<0.01	*Z* = −3.52	*p*<0.01
‘threshold−1’	35.0±5.3	4.3–82.6	*Z* = −7.09	*p*<0.0001	5.8±1.6	0.001–24.3	*Z* = −2.84	*p*<0.01	*Z* = −3.22	*p*<0.01
‘threshold’	5.3±3.1	0.002–60.5	*Z* = −0.47	*p* = 0.642	5.6±1.3	0.0–20.5	*Z* = −2.33	*p*<0.05	*Z* = −1.85	*p* = 0.064
‘threshold+1’	21.0±4.4	1.5–68.0	*Z* = −5.78	*p*<0.0001	6.9±1.8	0.04–20.8	*Z* = −2.95	*p*<0.01	*Z* = −2.33	*p*<0.05
‘threshold+2’	62.5±5.1	13.8–100.0	*Z* = −7.32	*p*<0.0001	2.3±0.7	0.0–9.8	*Z* = −1.58	*p* = 0.115	*Z* = −3.72	*p*<0.001

WRS: Wilcoxon rank sum test; WSR: Wilcoxon signed rank test.

Then, we compared explained variance of pin distances and explained variance of decision hysteresis between the current pin distance ‘threshold’ and each of the other four pin distances. For pairwise comparisons we used the Wilcoxon signed rank test. The test yielded significant differences for explained variance of pin distances between ‘threshold’ and ‘threshold−2’ (*Z* = −3.52, *p*<0.01), ‘threshold−1’ (*Z* = −3.34, *p*<0.01), ‘threshold+1’ (*Z* = −3.15, *p*<0.01) and ‘threshold+2’ (*Z* = −3.72, *p*<0.001), but no significant differences for the variance explained by decision hysteresis at threshold and the other current distances (see Table 3 for an overview).

**Table 3:** Statistics for Wilcoxon signed rank tests for the comparison of explained variance of pin distances and decision hysteresis at ‘threshold’ versus the other four current pin distances.

ExplainedVariance	Current PinDistance	Mean ± SEM (%)	Current PinDistance	Mean ± SEM (%)	WSR *z*-value	WSR *p*-value
Pin Distance	threshold	5.3±3.1	threshold−2	52.1±5.3	Z = −3.52	p<0.01
	threshold	5.3±3.1	threshold−1	35.0±5.3	Z = −3.34	p<0.01
	threshold	5.3±3.1	threshold+1	21.0±4.4	Z = −3.15	p<0.01
	threshold	5.3±3.1	threshold+2	62.5±5.1	Z = −3.72	p<0.001
Decision Hysteresis	threshold	5.6±1.3	threshold−2	3.2±0.9	Z = −1.81	p = 0.070
	threshold	5.6±1.3	threshold−1	5.8±1.6	Z = −0.24	p = 0.809
	threshold	5.6±1.3	threshold+1	6.9±1.8	Z = −0.89	p = 0.372
	threshold	5.6±1.3	threshold+2	2.3±0.7	Z = −2.46	p = 0.056

WSR: Wilcoxon signed rank test.

**Figure 4:**
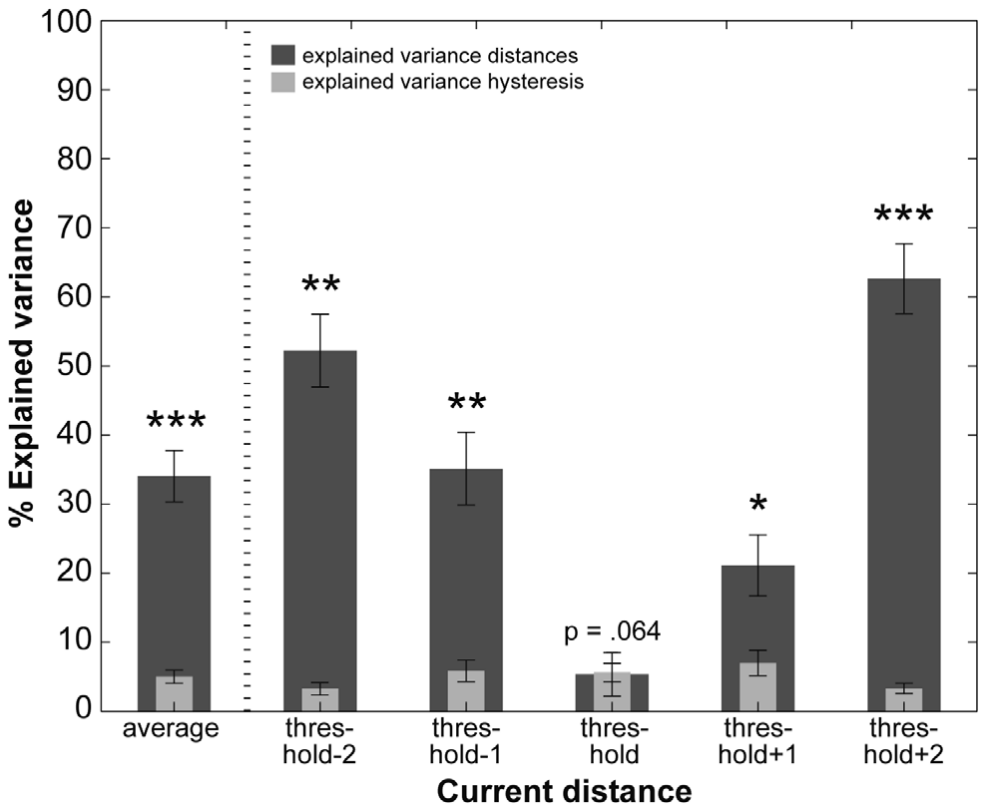
Explained variance of pin distances and decision hysteresis. Bar graphs show the amount of explained variance of participants’ decisions for stimulus property (i.e., presented pin distance; dark gray bars) and decision hysteresis (light gray bars). The left panel shows explained variances across all five current and preceding pin distances; the right panel shows explained variances for each of the currently presented pin distances in combination with all other pin distances. Whiskers represent the standard error of the mean. Significant differences between variances explained by pin distances and hysteresis are denoted with one star for *p*<0.05, with two stars for *p*<0.01 and with three stars for *p*<0.001. See also Table 2.

**Figure 5:**
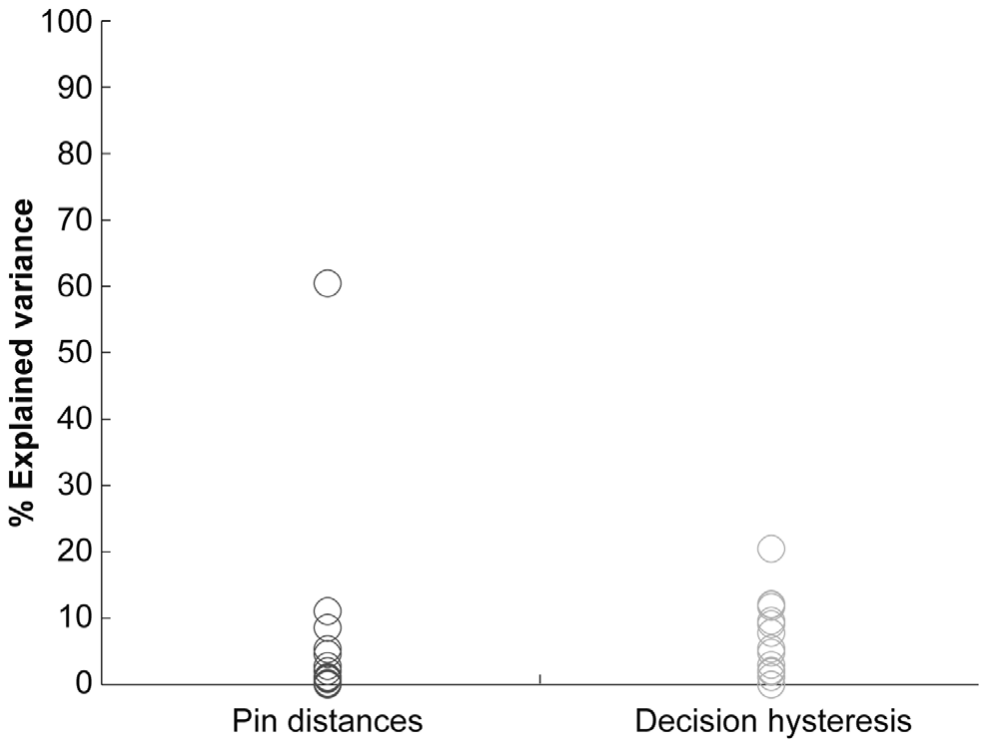
Explained variance of pin distances and decision hysteresis at threshold. The scatterplot shows explained variance of pin distances (dark gray circles) and decision hysteresis (light gray circles). At threshold we revealed a trend showing that hysteresis explains more variance than the stimulus (i.e., the applied pin distance).

Next, we directly compared the amount of variance explained by pin distances and decision hysteresis. Across all current pin distances the Wilcoxon signed rank test indicated that pin distances explained significantly more variance than hysteresis: *Z* = −3.78, *p*<0.001, see left panel of Figure 4. Post-hoc pairwise comparisons for each of the five current pin distances using Wilcoxon signed rank test yielded a significant difference for all current pin distances except for ‘threshold’ distances: ‘threshold−2’ (*Z* = −3.52, *p*<0.01), ‘threshold−1’ (*Z* = −3.22, *p*<0.01), ‘threshold+1’ (*Z* = −2.33, *p*<0.05), and ‘threshold+2’ (*Z* = −3.72, *p*<0.001) suggesting that the pin distance itself influenced participants’ decisions to a larger extend than hysteresis. At threshold, the Wilcoxon signed rank test yielded a trend for the inverse effect, which indicates that the variance of hysteresis had a more profound influence than the pin distances: *Z* = −1.85, *p* = 0.064, see Figure 5. Please note the large inter-individual differences in the magnitude of explained variance for both hysteresis and pin distances across participants (see right panel of Figure 4 and Table 2 for an overview).

### Correlation Analysis

**Figure 6:**
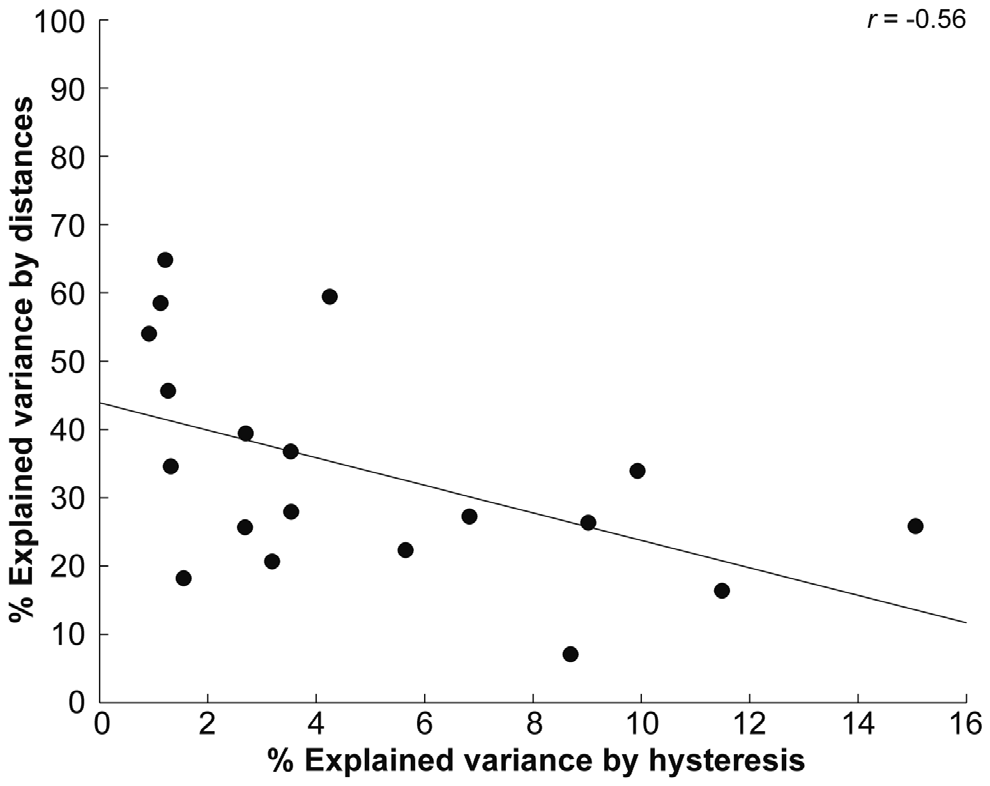
Spearman correlation analysis between explained variance of pin distances and decision hysteresis. The scatterplot shows the negative correlation between explained variance of pin distances and decision hysteresis. This suggests that the less variance was explained by the stimulus (i.e., the applied pin distance), the more influential hysteresis became for decisions.

We found a significant negative correlation between the amount of variance explained by pin distances and decision hysteresis: Spearman *R* = −0.56, *p*<0.05, see Figure 6. In other words, the less variance was explained by pin distances, the more variance was explained by hysteresis.

### Questionnaire

We asked participants if they had used a specific strategy when deciding on the presented pin distances. Seventeen participants responded that they did not use a strategy, one participant focused his attention on the stimulated finger, and one participant tried to visualize the pins. No participant claimed to have memorized the preceding decision.

## Discussion

In the present study we investigated whether hysteresis, describing the stability of a percept in spite of a change of physical stimulus properties [4], also leads to the stability of tactile spatial decisions across successively presented stimuli with changing stimulus properties.

In the context of perception, hysteresis has been known to occur close to or at sensory threshold [4], [14]. Gradually increasing stimulus intensity from non-discriminable to discriminable leads to a pop-out at stronger stimulus intensities (i.e., higher threshold) as compared to decreasing stimulus intensity from discriminable to non-discriminable (i.e., lower threshold). To account for such effects when determining a sensory threshold, in 1947 Georg von Békésy introduced a systematic stimulus presentation, known as the staircase method [5]. There are many forms of staircase procedures [31]. For example, one common approach is to begin the test with a high stimulus intensity which is easy to detect. The intensity is then gradually decreased until the test person indicates the drop-out. At this point the staircase ‘reverses’ and intensity is increased until the test person indicates the pop-out, triggering the next reversal. The intensities for the last of these ‘reversals’ are then averaged to determine the detection threshold.

In the present study we used the 2-point discrimination task, but with a fully balanced and hence unbiased order of stimulus presentations and assessed the stability of perceptual decision making across successive trials by means of conditional probabilities(i.e. decision hysteresis). To this end, we compared the probability of detecting a distance between the two pins on the current trial, given that the same or a different decision (i.e., no distance felt between pins) occurred on the trial before. We found that hysteresis influenced decisions, and participants were more likely to decide ‘same’ rather than ‘different’ on successively presented pin distances (see Figure 2). Across pin distances, the variance explained by decision hysteresis was distributed inversely u-shaped, with lowest influences on largest and shortest pin distances, and most profound influences on decisions at threshold or, in other words, at chance level (see light gray bars in Figure 4). Thus, participants were more likely to maintain a previous decision if stimuli offered decreasing sensory evidence. Looking at the decision history we also assessed up to which decision in the decision history a correlation between current and previous decision existed. We performed a regression analysis to address this question and found positive correlations between the decision on the current distance and decisions further back in history up to the 17^th^ decision back (see Figure 3).

We also compared the variance explained by decision hysteresis with that given by the explicit stimulus property ‘pin distance’. As mentioned above, we found an inverted u-shaped distribution of the variance explained by hysteresis across applied pin distances with the strongest influence on decisions around threshold (from below to above threshold, see light gray bars in Figure 4). For the explicit stimulus property ‘pin distance’ we found the inverted distribution (i.e., u-shaped) with greatest influence on largest and shortest pin distances, and lowest influence at threshold (see dark gray bars in Figure 4). When directly comparing the influence of both factors, we found that across all distances stimulus property explained significantly more variance of participants’ decisions than hysteresis (Figure 4). At threshold, the explained variance of pin distances was significantly lower as compared to the other four distances (i.e., ‘threshold−2’, ‘threshold−1’, ‘threshold+1’ ‘threshold+2’). Contrarily, the amount of variance explained by hysteresis at threshold did not significantly differ compared to each of the other current distances (see Table 3), but when pin distances were at threshold, we found a trend for hysteresis to explain more variance than the pin distances (Figure 5). This agrees with recent studies showing that the effect of hysteresis is especially pronounced when sensory information is weak [20] or ambiguous [22].

Our findings suggest that hysteresis, as a potentially implicit decision factor, becomes increasingly important when explicit stimulus property ‘pin distance’ provides decreasing evidence. We tested this hypothesis and found a significant negative correlation between the variance explained by pin distances and hysteresis (see Figure 6). The less variance was explained by pin distances, the more variance was explained by hysteresis. This relationship suggests that hysteresis is not simply a threshold phenomenon, but acts as a general prior in perceptual decision making which complements the decision on a stimulus (not only the stimulus per se, see results). After participants finished the 2-point discrimination task we asked them if they had used a specific strategy when deciding on the pin distances. No participant claimed to have memorized the preceding decision, suggesting that the preceding decision was implicitly and not explicitly memorized. This sort of perceptual prior is fundamentally different to other decision-related priors, such as those known to be involved in reward-guided decision making, which are explicitly and hence consciously shaped. It is assumed that reward history forms representations of expectations about decision outcomes and the revision of future expectations in the light of the prediction error (i.e., discrepancy between actual outcome and prior expectation) [32], [33].

Implicitly formed prior information, as described by hysteresis, seems to contribute to perceptual decision making, most likely for complementing the decision process if the stimulus offers insufficient sensory evidence. This fits the free-energy principle postulated by Karl Friston [34]. The ensuing scheme rests on Empirical Bayes and hierarchical models of how sensory input is caused. The use of hierarchical models enables the brain to construct prior expectations in a dynamic and context-sensitive fashion [35]. In the context of hysteresis, our present findings suggest that for each presented pin distance the brain sustains the decision on that pin distance (rather than just the pin distance itself, see Results for further details) in order to complement the following decision process when sensory information is sparse. Importantly, for threshold stimuli the empirical evidence for either of the decisions is not sufficiently strong to clearly select one of the two options. According to the free-energy principle, staying with the same decision would maintain the status quo associated with minimising surprise. Thus, staying with the same decision should be most evident for threshold stimuli, which we revealed in the present study. This process is obviously more efficient than evaluating each pin distance independently from any prior information, especially when sensory information is weak [20] or ambiguous [22]. It is rather surprising that hysteresis in the present study only accounted for up to 5.8 to 6.9% of the decision variance (see Table 2; ‘threshold−1’, ‘threshold’, ‘threshold+1’). Previous studies, which quantified the influence of the time-order effect on perceptual decision making in a delayed discrimination task (see Introduction), assigned 57% of the variance to this sort of prior information [9]. This difference in explained variance of both priors suggests, that either delayed discrimination tasks involve more ambiguous stimulus information due to the required memorizing of stimuli, or that the unbiased and balanced stimulus presentation (as compared to strictly ascending and descending stimulus sequences) may have attenuated the influence of hysteresis on perceptual decision making. Such a presentation schema obviously involves more pronounced trial-to-trial differences in stimulus properties as compared to strictly ascending or descending orderings. In these latter presentation orders the direction of stimulus contrast is constant for consecutive stimuli, which results in high resemblance and high predictability of stimuli, thus supporting carryover effects such as decision hysteresis. The unsystematic presentation order may thus have amplified the influence of stimulus properties to the disadvantage of the hysteresis effect. This assumption is also supported by the study by Hock and colleagues [17]. They presented ambiguous visual stimuli in a random order as well as in ascending and descending orders. Bistability was observed for fewer stimuli when they were randomly presented. With a sequential presentation of stimuli, bistability was indicated over almost the entire range of stimuli [17].

The explicit factor of interest (i.e., pin distance) explained from 52.1 to 62.5% (see Table 2; ‘threshold−2’, ‘threshold+2’) of the decision variance. Adding the variance explained by hysteresis suggests that other factors such as attention, task instructions, and motivation may additionally contribute to the perceptual decision process [2], which have to be considered in future studies.

## Conclusions

In the present study we first examined the influence of hysteresis on the stability of perceptual decisions across tactile spatial stimuli, presented in a fully balanced and hence unbiased way with regard to the trial-to-trial order of pin distances. We showed that participants were more likely to decide ‘same’ rather than ‘different’ on two successively presented pin distances. This effect was not only significant at threshold but was even present for easy to discriminate pin distances (see Figure 2). By means of a nonparametric regression analysis, we found a positive correlation between the current decision and decisions further back in the decision history for up to 17 decisions back (see Figure 3).

Second, we quantified to what extent stimulus property (i.e., the applied pin distance) and hysteresis influenced participants’ decisions by means of explained variances. Across stimuli presentations the applied pin distances explained significantly more variance of participants’ decisions, but we found the strongest influence of hysteresis when sensory evidence was maximally weak. This was especially true for pin distances presented at threshold. For those decisions, our results revealed a trend showing that hysteresis explained more variance of participants’ decision than the stimulus properties (i.e., pin distance) (see Figure 4 and 5).

Explained variances of hysteresis and pin distances correlated negatively, suggesting that if less variance was explained by pin distances (e.g., at threshold), then more variance was explained by hysteresis (see Figure 6). These results agree with the notion that hysteresis acts as prior information to stabilize percepts in noisy and unstable environments [14].

## References

[1] Heekeren HR, Marrett S, Ungerleider LG (2008) The neural systems that mediate human perceptual decision making. Nat Rev Neurosci 9: 467–479. 18464792 10.1038/nrn2374

[2] Ratcliff R, McKoon G (2008) The diffusion decision model: theory and data for two-choice decision tasks. Neural Comput 20: 873–922. 18085991 10.1162/neco.2008.12-06-420 PMC2474742

[3] Gold JI, Shadlen MN (2007) The neural basis of decision making. Annu Rev Neurosci 30: 535–574. 17600525 10.1146/annurev.neuro.29.051605.113038

[4] Sekuler R (1996) Motion perception: A modern view of Wertheimer’s 1912 monograph. Perception 25: 1243–1258. 9027927 10.1068/p251243

[5] Von Békésy G (1947) A New Audiometer. Acta Otolaryngol 35: 411–422.

[6] Romo R, de Lafuente V (2013) Conversion of sensory signals into perceptual decisions. Prog Neurobiol 103: 41–75. 22472964 10.1016/j.pneurobio.2012.03.007

[7] Romo R, Salinas E (2003) Flutter discrimination: neural codes, perception, memory and decision making. Nat Rev Neurosci 4: 203–218. 12612633 10.1038/nrn1058

[8] Sinclair RJ, Burton H (1996) Discrimination of vibrotactile frequencies in a delayed pair comparison task. Percept Psychophys 58: 680–692. 8710447 10.3758/bf03213100

[9] Karim M, Harris JA, Morley JW, Breakspear M (2012) Prior and present evidence: how prior experience interacts with present information in a perceptual decision making task. PLoS One 7: e37580. 22701521 10.1371/journal.pone.0037580 PMC3362626

[10] Preuschhof C, Schubert T, Villringer A, Heekeren HR (2010) Prior Information biases stimulus representations during vibrotactile decision making. J Cogn Neurosci 22: 875–887. 19413475 10.1162/jocn.2009.21260

[11] Hellström A (1985) The time-order error and its relatives: Mirrors of cognitive processes in comparing. Psychol Bull 97: 1161–1177.

[12] Helson H (1964) Adaptation-level theory. New York: Harper & Row.

[13] Fechner GT (1860) Elemente der Psychophysik. Leipzig, Germany: Breitkopf & Härtel.

[14] Kleinschmidt A, Büchel C, Hutton C, Friston KJ, Frackowiak RSJ (2002) Expressing Perceptual Hysteresis in Visual Letter Recognition. Neuron 34: 659–666. 12062048 10.1016/s0896-6273(02)00694-3

[15] Gepshtein S, Kubovy M (2005) Stability and change in perception: spatial organization in temporal context. Exp brain Res 160: 487–495. 15517224 10.1007/s00221-004-2038-3

[16] Schwiedrzik CM, Ruff CC, Lazar A, Leitner FC, Singer W, et al.. (2012) Untangling Perceptual Memory: Hysteresis and Adaptation Map into Separate Cortical Networks. Cereb Cortex. 10.1093/cercor/bhs396 PMC3977616 23236204

[17] Hock HS, Kelso JA, Schöner G (1993) Bistability and hysteresis in the organization of apparent motion patterns. J Exp Psychol Hum Percept Perform 19: 63–80. 8440989 10.1037//0096-1523.19.1.63

[18] Hock HS, Bukowski L, Nichols DF, Huisman A, Rivera M (2005) Dynamical vs. judgmental comparison: hysteresis effects in motion perception. Spat Vis 18: 317–335. 16060230 10.1163/1568568054089393

[19] Melloni L, Schwiedrzik CM, Müller N, Rodriguez E, Singer W (2011) Expectations change the signatures and timing of electrophysiological correlates of perceptual awareness. J Neurosci 31: 1386–1396. 21273423 10.1523/JNEUROSCI.4570-10.2011 PMC6623627

[20] Brady TF, Oliva A (2012) Spatial frequency integration during active perception: perceptual hysteresis when an object recedes. Front Psychol 3: 462. 23162509 10.3389/fpsyg.2012.00462 PMC3498875

[21] Odic D, Hock H, Halberda J (2012) Hysteresis Affects Approximate Number Discrimination in Young Children. J Exp Psychol Gen. 10.1037/a0030825 PMC4390026 23163765

[22] Sacharin V, Sander D, Scherer KR (2012) The perception of changing emotion expressions. Cogn Emot 26: 1273–1300. 22550942 10.1080/02699931.2012.656583

[23] Raczaszek J, Tuller B, Shapiro LP, Case P, Kelso S (1999) Categorization of ambiguous sentences as a function of a changing prosodic parameter: a dynamical approach. J Psycholinguist Res 28: 367–393. 10380661 10.1023/a:1023289031747

[24] Schütz C, Weigelt M, Odekerken D, Klein-Soetebier T, Schack T (2011) Motor control strategies in a continuous task space. Motor Control 15: 321–341. 21878687 10.1123/mcj.15.3.321

[25] Weigelt M, Rosenbaum D a, Huelshorst S, Schack T (2009) Moving and memorizing: motor planning modulates the recency effect in serial and free recall. Acta Psychol (Amst) 132: 68–79. 19591968 10.1016/j.actpsy.2009.06.005

[26] Wichmann F, Hill N (2001) The psychometric function: I. Fitting, sampling, and goodness of fit. Percept Psychophys 63: 1293–1313. 11800458 10.3758/bf03194544

[27] Dinse HR, Ragert P, Pleger B, Schwenkreis P, Tegenthoff M (2003) Pharmacological modulation of perceptual learning and associated cortical reorganization. Science (80-) 301: 91–94. 10.1126/science.1085423 12843392

[28] Pleger B, Dinse HR, Ragert P, Schwenkreis P, Malin J, et al (2001) Shifts in cortical representations predict human discrimination improvement. Proc Natl Acad Sci U S A 98: 12255–12260. 11593042 10.1073/pnas.191176298 PMC59801

[29] Pleger B, Foerster A, Ragert P, Dinse HR, Schwenkreis P, et al (2003) Functional imaging of perceptual learning in human primary and secondary somatosensory cortex. Neuron 40: 643–653. 14642286 10.1016/s0896-6273(03)00677-9

[30] Rasmussen CE, Williams CK (2006) Gaussian Processes for Machine Learning. MIT Press.

[31] García-Pérez M (2000) Optimal setups for forced-choice staircases with fixed step sizes. Spat Vis 13: 431–448. 11310536 10.1163/156856800741306

[32] Rushworth MFS, Mars RB, Summerfield C (2009) General mechanisms for making decisions? Curr Opin Neurobiol 19: 75–83. 19349160 10.1016/j.conb.2009.02.005

[33] Lau B, Glimcher PW (2005) Dynamic Response-by-Response Models of Matching Behavior in Rhesus Monkeys. J Exp Anal Behav 84: 555–579. 16596980 10.1901/jeab.2005.110-04 PMC1389781

[34] Friston K (2010) The free-energy principle: a unified brain theory? Nat Rev Neurosci 11: 127–138. 20068583 10.1038/nrn2787

[35] Friston K, Kilner J, Harrison L (2006) A free energy principle for the brain. J Physiol Paris 100: 70–87. 17097864 10.1016/j.jphysparis.2006.10.001

